# PTC-MAS: A Deep Learning-Based Preoperative Automatic Assessment of Lymph Node Metastasis in Primary Thyroid Cancer

**DOI:** 10.3390/diagnostics13101723

**Published:** 2023-05-12

**Authors:** Ruqian Fu, Hao Yang, Dezhi Zeng, Shuhan Yang, Peng Luo, Zhijie Yang, Hua Teng, Jianli Ren

**Affiliations:** 1 Department of Ultrasound, The Second Affiliated Hospital of Chongqing Medical University, Chongqing 400010, China; 2 Medical Data Science Academy, Chongqing Medical University, Chongqing 400010, China; 3 Breast & Thyroid Surgery, The Second Affiliated Hospital of Chongqing Medical University, Chongqing 400010, China

**Keywords:** transfer learning, lymph node metastasis, thyroid cancer, deep learning, ultrasonography, diagnosis

## Abstract

Background: Identifying cervical lymph node metastasis (LNM) in primary thyroid cancer preoperatively using ultrasound is challenging. Therefore, a non-invasive method is needed to assess LNM accurately. Purpose: To address this need, we developed the Primary Thyroid Cancer Lymph Node Metastasis Assessment System (PTC-MAS), a transfer learning-based and B-mode ultrasound images-based automatic assessment system for assessing LNM in primary thyroid cancer. Methods: The system has two parts: YOLO Thyroid Nodule Recognition System (YOLOS) for obtaining regions of interest (ROIs) of nodules, and LMM assessment system for building the LNM assessment system using transfer learning and majority voting with extracted ROIs as input. We retained the relative size features of nodules to improve the system’s performance. Results: We evaluated three transfer learning-based neural networks (DenseNet, ResNet, and GoogLeNet) and majority voting, which had the area under the curves (AUCs) of 0.802, 0.837, 0.823, and 0.858, respectively. Method III preserved relative size features and achieved higher AUCs than Method II, which fixed nodule size. YOLOS achieved high precision and sensitivity on a test set, indicating its potential for ROIs extraction. Conclusions: Our proposed PTC-MAS system effectively assesses primary thyroid cancer LNM based on preserving nodule relative size features. It has potential for guiding treatment modalities and avoiding inaccurate ultrasound results due to tracheal interference.

## 1. Introduction

Thyroid cancer is an increasingly important topic in public health due to its rising incidence in high- and middle-income countries [1,2]. According to recent estimates [1,2], thyroid cancer now accounts for 3% of all cancers worldwide. Current guidelines [3] recommend ultrasound assessment of thyroid nodules and cervical lymph nodes for all patients with confirmed or suspected thyroid nodules. Ultrasound imaging is preferred over other imaging modalities because it provides more accurate representations of the anatomy and allows for real-time monitoring of changes in lymph nodes. Furthermore, ultrasound is the imaging modality of choice for the evaluation of cervical lymph node metastasis (LNM), enabling identification and characterization of abnormal central and lateral cervical lymph nodes, thereby facilitating surgical management [3,4].

The American Joint Committee on Cancer (AJCC) [5] grouping method is widely used in surgical and oncological settings for diagnosing and treating cervical LNM. AJCC classifies lymph nodes in the neck into groups I–VII [5], based on the extent and level of involvement. In cases of thyroid cancer, the central compartment (group VI) between the lower cricoid cartilage and the supraclavicular fossa is commonly the first site of LNM [3,6]. To differentiate between benign and malignant lymph nodes, grey-scale ultrasound is typically used to evaluate size, shape, margins, hilum, and nodal echogenicity, whereas color Doppler ultrasound examines vascular location and impedance values [7]. However, assessing cervical lymph nodes by grey-scale and color Doppler ultrasound using AJCC lymphatic subdivisions is a subjective and labor-intensive procedure that often results in low sensitivity (25–60%) [8,9,10]. This, in turn, increases the risk of prophylactic lymph node dissection in low-risk thyroid cancer patients without LNM, which can lead to complications such as hypoparathyroidism and laryngeal nerve dysfunction [9]. Therefore, preoperative assessment of cervical lymph nodes is essential in patients with thyroid cancer [11,12], as ultrasound examination can be challenging, even for experienced radiologists, due to interference from gas in the trachea and esophagus, as well as the varying degrees of expertise among radiologists [6].

LNM risk, which includes the risk of recurrence, distant metastases, and disease-specific mortality, is a critical factor to consider in the management of thyroid cancer [13,14,15]. Traditional statistical analysis has been used in several studies [16,17,18,19,20] to evaluate LNM risk based on factors such as tumor size, patient age, extrathyroidal invasion, vascular invasion, microcalcification, and concomitant Hashimoto’s disease. Of these, tumor size has been identified as an independent risk factor for LNM. Advancements in computer hardware have facilitated the development of computer-aided systems to diagnose LNM in thyroid cancer accurately. Researchers [21,22] have explored the use of radiomics to analyze thyroid ultrasound images. Radiomics involves extracting high-throughput features such as textures, boundaries, and wavelets from thyroid ultrasound images to construct models for thyroid cancer LNM. However, the performance of these models for detecting LNM based on ultrasound sweeps of cervical lymph nodes, as measured by the area under the curves (AUCs) on an independent test set, has been found to be only 0.64 to 0.80 [23,24,25,26], indicating that the predictive power of these models is still limited. This is due to the low positive rate of ultrasound-based cervical lateralization, as the sensitivity and specificity of ultrasound for assessing cervical LNM are not high, and ultrasound results are highly dependent on the operator’s diagnostic experience. Furthermore, radiomics requires radiologists to manually extract multiple features after selecting regions of interest (ROIs), which can be a labor-intensive and biased process [27,28,29].

With the rapid development of artificial intelligence, deep learning-based diagnostic models have gained significant attention due to their ability to achieve diagnostic accuracy comparable to doctors in many areas [30,31,32]. To evaluate the risk of LNM in thyroid cancer, various clinical features were introduced into machine learning algorithms to develop LNM assessment models, such as those developed by Zhu et al. [33] and Zou et al. [34]. Additionally, Wu et al. [35] and Liu et al. [36] employed conventional univariate and multivariate analyses on the collected data regarding multiple clinical characteristics and ultrasonography features, and machine learning algorithms were constructed. Wu et al. [37] further explored the information contained in ultrasound images and combined features extracted from B-mode and color Doppler flow imaging ultrasound images with clinical data to construct a multimodal assessment model using InceptionResnetV2, which achieved a better assessment result. Moreover, Zou et al. [38] explored the combination of multiple forms of medical images and constructed an XGBoost model based on ultrasound and dual-energy computed tomography images of solitary primary lesions. However, the above methods required radiologists to manually extract ROIs for each ultrasound image, making feature extraction subjective and poorly reproducible. Additionally, combining different types of images can complement each other to improve the model’s performance, but it also raised the threshold for using the model as it requires multiple images of the patient to be taken simultaneously.

The objective of this study was to develop a deep learning system using B-mode ultrasound images of thyroid lesions to predict the presence of LNM in patients with thyroid cancer. By using a more objective and reproducible feature extraction process, we aim to improve the accuracy of LNM assessment and reduce the risk of prophylactic lymph node dissection in low-risk thyroid cancer patients without LNM. Ultrasound sweeps of cervical LNM have low sensitivity and specificity, so we chosed to use ultrasound images of thyroid cancer for indirect assessment of cervical LNM. This approach avoided interference from intratracheal and esophageal gases during direct assessment and reduced the influence of radiologist expertise in assessing cervical lymph nodes. Our system was designed to automatically extract thyroid nodules from ultrasound images and output a prediction of LNM.

## 2. Materials

**Figure 1:**
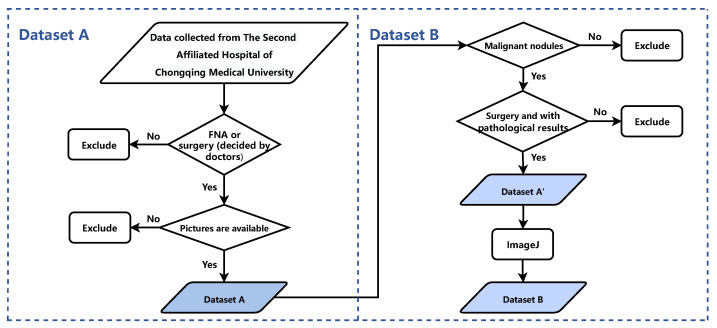
The process of the patient enrollment for Dataset A and Dataset B. FNA: Fine Needle Aspiration.

This study relied on two datasets: Dataset A and Dataset B. Dataset A was used to develop the YOLO [39] Thyroid Nodule Recognition System (YOLOS), while Dataset B was employed for the Primary Thyroid Cancer Lymph Node Metastasis Assessment System (PTC-MAS). Notably, Dataset B is a subset of Dataset A, created by extracting a portion of the data from the latter. Figure 1 outlines the data screening process, with the left side depicting the patient enrollment process for Dataset A and the right side illustrating the steps involved in creating Dataset B. The figure provides a clear overview of the relationship between the two datasets.

Dataset A comprises 2431 thyroid nodules extracted from ultrasound images of patients who received treatment at the Second Affiliated Hospital of Chongqing Medical University (SAHCMU) between June 2018 and February 2022. The dataset was created by applying the following inclusion criteria: (1) thyroid nodules with clear B-mode ultrasound images; (2) thyroid nodules confirmed by either fine-needle aspiration (FNA) or thyroidectomy; and (3) FNA or surgery performed within 30 days of ultrasound imaging. The exclusion criteria were as follows: (1) reports of ultrasound findings that did not correspond to the reports of pathological findings in terms of location or size; (2) measuring lines visible on the ultrasound images; and (3) patients who had received preoperative treatments such as chemotherapy, radiotherapy, or hormone therapy.

Dataset B is a subset of Dataset A that comprises 1002 malignant thyroid nodules identified from ultrasound images. To create Dataset B, Dataset A’ was first curated by excluding nodules from Dataset A that met one or more of the following criteria: (1) FNA biopsy only, without subsequent surgical intervention; (2) nodules with benign pathology; (3) multifocal lesions with incomplete lymph node data; and (4) images with excessive enlargement or reduction. Then, an ultrasound image from Dataset A’ was used as a standard to align the scale of all ultrasound images using ImageJ (https://imagej.nih.gov/ij/, accessed on 25 February 2022), resulting in normalized images as Dataset B. Table 1 summarizes patient demographics, tumor size, invasiveness of operations, American College of Radiology (ACR) Thyroid Imaging Reporting and Data System (TI-RADS) classification, and the number of lymph nodes for malignant nodules in both datasets.

This study used ultrasound machines manufactured by GE Healthcare (LOGIQ E9, LOGIQ S7), Samsung (RS80A), Mindray (Resona 7T), and Philips (EPIQ5, EPIQ7, IU22, IU elite) to obtain thyroid images. The images were retrieved from the Picture Archiving and Communication System (PACS) workstation of the SAHCMU in JPEG format. A total of eight operators acquired the images using frequencies ranging from 8 to 13 MHz.

All thyroid nodules that met the inclusion criteria underwent either FNA or surgery, and were pathologically diagnosed by two expert pathologists. The retrospective study received approval from the Ethics Committee, and patient informed consent was waived.

**Table 1:** Baseline characteristics of Dataset A and Dataset B.

Characteristics	Dataset A (YOLOS)		Dataset B (PTC-MAS)	
	Nodules		LNM Status	
	Benign	Malignant	Yes	No
Age (y)	49 ± 13	43 ± 12	37 ± 12	44 ± 12
Sex				
Female	1021 (81.3%)	873 (74.3%)	286 (64.9%)	459 (81.8%)
Male	235 (18.7%)	302 (25.7%)	155 (35.1%)	102 (18.2%)
Tumor size (cm)				
≤0.5	140 (11.1%)	218 (18.6%)	25 (5.7%)	121 (21.6%)
0.5–1.0	270 (21.5%)	593 (50.5%)	183 (41.5%)	318 (56.7%)
1.0–2.0	301 (24.0%)	267 (22.7%)	161 (36.5%)	100 (17.8%)
>2.0	545 (43.4%)	97 (8.3%)	72 (16.3%)	21 (3.7%)
TI-RADS				
2	25 (2.0%)	0 (0.0%)	\	\
3	566 (45.1%)	20 (18.2%)	4 (0.9%)	3 (0.5%)
4A	334 (26.6%)	84 (7.1%)	21 (4.8%)	39 (7.0%)
4B	223 (17.8%)	345 (29.4%)	124 (28.1%)	183 (32.6%)
4C	105 (8.4%)	670 (57.0%)	246 (55.8%)	324 (57.8%)
5	3 (0.2%)	56 (4.8%)	46 (10.3%)	12 (2.1%)
FNA	301 (24.0%)	30 (2.6%)	0 (0%)	0 (0%)
Surgery	955 (76.0%)	1145 (97.4%)	441 (100%)	561 (100%)
No. of LN				
<3	\	\	49 (11.1%)	196 (34.9%)
3–5	\	\	74 (16.8%)	166 (29.6%)
≥5	\	\	318 (72.1%)	199 (35.5%)
Total	1256	1175	441	561

YOLOS: YOLO Thyroid Nodule Recognition System, PTC-MAS: Primary Thyroid Cancer Lymph Node Metastasis Assessment System, LNM: Lymph Node Metastasis, FNA: Fine Needle Aspiration, LN: Lymph Nodes.

## 3. Methods

**Figure 2:**
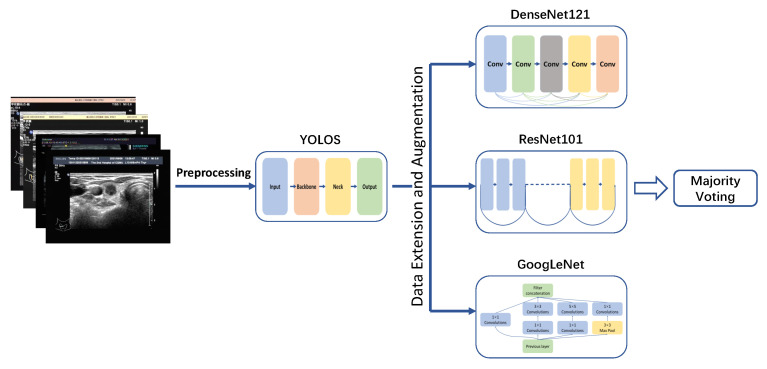
Lymph node metastases recognition models development. YOLOS: YOLO Thyroid Nodule Recognition System.

In this section, we described the architecture of PTC-MAS (Figure 2), which consisted of two main components: automatic ROIs extraction and LNM classification probability assessment. The first component utilized YOLOS to extract ROIs from ultrasound images. In the second component, the ROIs generated by YOLOS were fed into three separate networks (DenseNet121 [40], ResNet101 [41], and GoogLeNet [42]) to obtain classification results. These results were then combined using a voting classification method to generate the final assessment result.

To build this system, we used a detailed process that involved selecting appropriate parameters and optimizing the performance of each component. We have provided a comprehensive description of this process in the following sections.

### 3.1. Image Preprocessing

Ultrasound images obtained from PACS often contain noise, such as ultrasound device parameters, that can interfere with the performance of neural networks when used for image classification tasks. To address this issue, we implemented a manual noise removal step using Labelme (version 3.16.2, https://github.com/wkentaro/labelme, accessed on 24 January 2022) by radiologists. During this step, the radiologists carefully removed noisy information surrounding the raw ultrasound images while preserving the tissue structure images to the greatest extent possible. The resulting images, which were free of noise information, were used to train the YOLOS. To ensure the high quality of the dataset, each image was thoroughly reviewed by the radiologist to ensure that that it met the inclusion criteria before being used for classification.

### 3.2. Extraction of ROIs Using YOLOS

We present YOLOS, a novel automated approach for extracting target nodal ROIs from ultrasound images using YOLOv5 (https://github.com/ultralytics/yolov5, accessed on 18 January 2022) object detection model with adaptive anchor boxes. The primary objective of YOLOS is to streamline radiologists’ workload and provide a standardized, reproducible methodology for ROIs extraction. To ensure the accuracy of our extracted ROIs, we employed an experienced radiologist with over 5 years of experience to manually annotate them using Labelme (version 3.16.2, https://github.com/wkentaro/labelme, accessed on 24 January 2022). These annotated images were then reviewed and adjusted by a senior radiologist with more than 10 years of experience, serving as the gold standard for evaluation.

We employed various data augmentation techniques, such as mosaic [39], horizontal flipping, and random scaling, on the training set. We fine-tuned the hyperparameters of YOLOS using a genetic algorithm [43] to optimize its overall performance.

### 3.3. Assessment of LNM Status

#### 3.3.1. Image Cropping Methods

**Figure 3:**
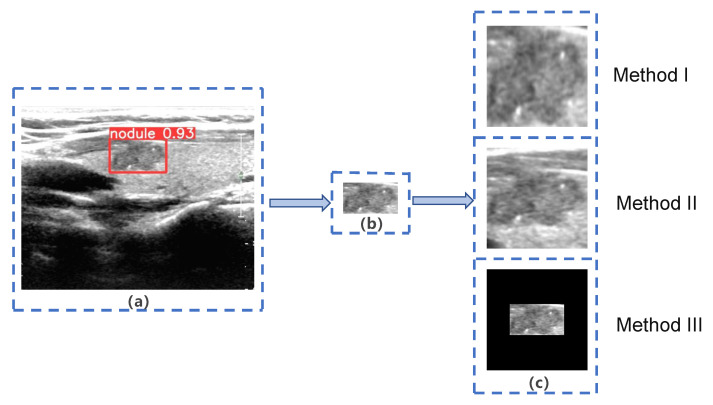
Three different methods of image extension. (**a**) Target nodule marked by YOLO Thyroid Nodule Recognition System(YOLOS); (**b**) Region of Interest (ROI) extracted by YOLOS; (**c**) three different methods of image extension.

**Figure 4:**
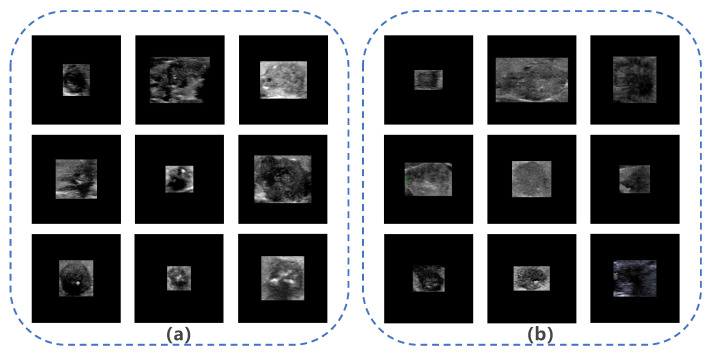
Images of nodules with and without lymph node metastasis (LNM). (**a**) Example images of nodules with LNM; (**b**) example images of nodules without LNM.

The ROIs extracted from malignant thyroid nodule images in Dataset B by YOLOS were used as input for subsequent networks. However, network inputs are typically fixed at 224 × 224 pixels. Previous studies [44,45] have identified two main methods (Method I and Method II) for resizing images, as shown in Figure 3. Method I directly resizes ROIs to the target size, but this method changes the nodules’ scale, making them significantly different from real nodules, such as changes in the aspect ratio and shape. Therefore, we decided not to use Method I, as it is not in alignment with clinical practice [3]. Method II extends the ROIs to a square with the largest diameter of the ROIs as the side length, preserving the scale and surrounding tissue structure of the nodule but ignoring important information about the nodule’s size. However, based on existing clinical studies [16,17,18,19], identifying nodule size as an independent risk factor for LNM, we placed the ROIs in the center of a fixed-size square patch with zero padding (Method III). This approach preserves the nodule’s morphological proportions and size, but missing some of the surrounding tissue structure. Figure 4 displays images of nodules with and without LNM, both processed using Method III. We hypothesized that Method III can improve the system’s performance by providing more accurate information about nodule size and the aspect ratio, which is an important risk factor for LNM.

#### 3.3.2. Assessment of LNM Probability

The first step of our system involved using YOLOS to extract ROIs. These ROIs were then fed into three different neural networks in the second part of the system: DenseNet, ResNet, and GoogLeNet. We selected these networks for their high efficiency, accuracy, and relatively low number of parameters. DenseNet reduces network parameters by reusing features and bypassing the network to alleviate the vanishing gradient problem. ResNet uses a residual structure to construct an ultra-deep network and accelerates training through batch normalization. GoogLeNet employs an inception structure to fuse features from different scales, reduces model parameters through an average pooling layer, and utilizes a 1 × 1 convolution kernel for dimensionality reduction. Each network was trained separately on the same dataset, and produced classification results of ROIs independently.

To integrate the ROIs classification results from these three networks, we employed the majority voting method, which combines the results of multiple networks and achieves the learning task by following the majority rule, thereby reducing variance and improving network robustness. We used soft voting in our system, which aggregates the classification results through majority voting to obtain the final assessment result.

#### 3.3.3. Parameters of the Three Networks

In this study, we evaluated two different image cropping methods, Method II and Method III, by feeding the corresponding ROIs into three neural networks. To expedite convergence, we initialized the network weights using pretraining on the ImageNet dataset (http://image-net.org/, accessed on 12 March 2022), which comprises approximately 15 million images. Transfer learning from this pretrained model can effectively mitigate the issue of limited medical data.

To train the three networks for assessing the result of LNM, we extracted ROIs from Dataset B using YOLOS and split them into a training set (90%) and a test set (10%). We performed various data augmentations on the training set, including cutmix [46], horizontal flipping, and brightness adjustments, to enhance the network’s ability to generalize to new data. To prevent overfitting, we used five-fold cross-validation for each network. We also employed five-fold cross-validation on the training set to prevent overfitting.

We optimized the networks using the AdamW [47] optimizer with a learning rate of 0.0001 and a weight decay of 0.003. We used cross-entropy loss as the objective function and cosine annealing to adjust the learning rate, which helped the system to escape local minima and find the global minimum. We evaluated the network parameters obtained from each cross-validation on the test set.

To compare the effects of different image cropping methods, we kept the model parameters consistent and varied the number of epochs for the models. We also used class activation maps (CAM) [48] to generate heatmaps that indicate the regions of the input images that are most relevant for the convolutional neural network (CNN) model’s prediction of LNM.

We implemented all models in PyTorch (version 1.9.1) on a computer with a GeForce RTX 3060 graphics processing unit (NVIDIA, Santa Clara, CA, USA) and a Core i7-11800H central processing unit (Intel, Santa Clara, CA, USA).

## 4. Results

In this section, we conducted several experiments to investigate the performance of PTC-MAS, the impact of different image cropping methods on PTC-MAS’s performance, and the accuracy of automatic ROIs extraction using YOLOS.

### 4.1. Evaluation Criteria

To assess the effectiveness of PTC-MAS, we used Youden’s J statistic to determine the optimal classification thresholds (Youden’s index), which were then applied to categorize the system output probabilities into true positives (TP), false positives (FP), true negatives (TN), and false negatives (FN). TP and TN indicate the number of correctly classified positive and negative samples, while FP and FN indicate the number of misclassified negative and positive samples. In this study, positive samples referred to nodules with LNM. We also plotted receiver operating characteristic (ROC) curves and calculated AUCs to evaluate the overall performance of the models on the test set. To determine whether there were significant differences in LNM assessments among the different models, we used DeLong’s test. Additionally, we used quantitative indices such as the $F_{1}$ score, sensitivity, accuracy, specificity, positive predictive value (PPV), and negative predictive value (NPV) to further evaluate the model’s performance. These evaluation indices are defined as follows:

(1) $$F_{1}=2\times \frac{precision\times recall}{precision+recall}$$

(2) $$accuracy=\frac{TP+TN}{TP+TN+FP+FN}$$

(3) $$specificity=\frac{TN}{TN+FP}$$

(4) $$sensitivity=\frac{TP}{TP+FN}$$

(5) $$PPV=\frac{TP}{TP+FP}$$

(6) $$NPV=\frac{TN}{TN+FN}$$

We calculated the 95% confidence interval (CI) using Wilson’s method and considered a two-tailed *p*-value less than 0.05 to be statistically significant. We performed all statistical analyses using MedCalc (version 20.109, Ostend, Belgium), SPSS (version 25.0, IBM, Chicago), and VassarStats (http://vassarstats.net/, accessed on 15 August 2022).

### 4.2. YOLOS Automatic ROIs Extraction Performance

**Figure 5:**
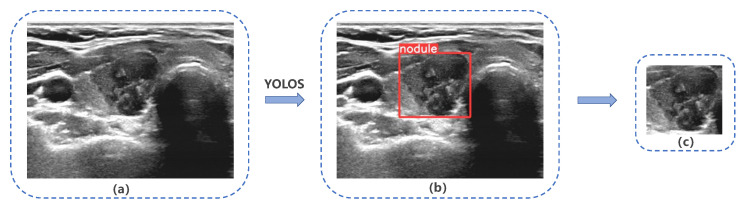
YOLO Thyroid Nodule Recognition System(YOLOS) detection and cropping of thyroid nodules in ultrasound images. (**a**) Original ultrasound image, (**b**) and (**c**) depict YOLOS-identified and cropped nodules, respectively.

Our study evaluated the performance of YOLOS on a test set consisting of 243 nodules, which were randomly selected from Dataset A, as shown in Figure 5. The YOLOS initially identified the presence of a nodule within the image and subsequently determined its bounding box coordinates. Based on these coordinates, YOLOS crops the ROI containing the nodule. In this test set, YOLOS achieved a precision of 0.999 and a sensitivity of 0.992.

Our findings suggested that YOLOS was highly accurate in extracting target nodal ROIs from ultrasound images, potentially reducing the time and effort required for radiologists to manually identify and annotate these regions. This efficiency could improve the consistency and reliability of ultrasound image analysis.

### 4.3. Thyroid Cancer LNM Recognition Model Performance

To evaluate the performance of PTC-MAS and compare majority voting with single models, we preprocessed the ROIs outputted by YOLOS based on Dataset B using Method III and trained individual models separately. We fine-tuned the initial weights of our models using transfer learning and pre-trained them on ImageNet. To ensure the stability of the system during training, we calculated the accuracy of the five-fold cross-validation of Method III on the training set of Dataset B. As shown in Table 2, the mean values of the five-fold cross-validation accuracy for each model ranged from 0.849 to 0.878.

**Table 2:** Five-fold cross-validation accuracy of Method III for different methods based on Dataset B.

	Fold 1	Fold 2	Fold 3	Fold 4	Fold 5	Average
DenseNet	0.864	0.849	0.885	0.892	0.887	0.875
ResNet	0.850	0.875	0.872	0.829	0.821	0.849
GoogLeNet	0.860	0.897	0.858	0.899	0.878	0.878

**Figure 6:**
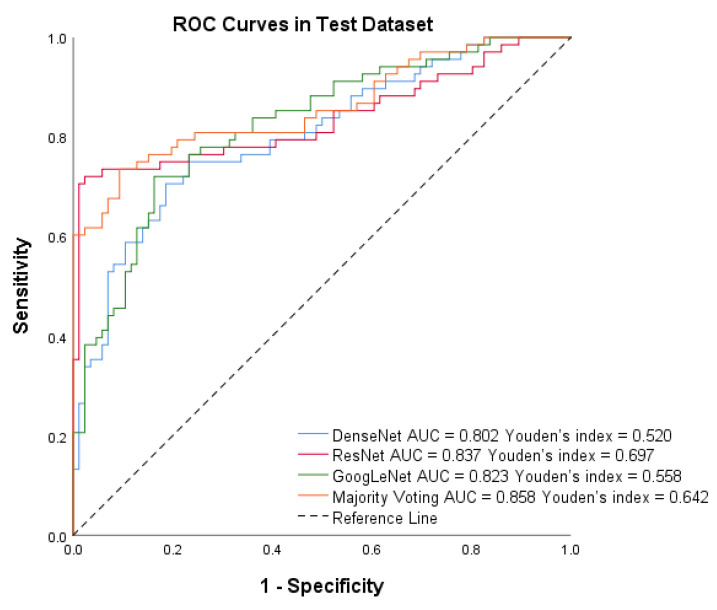
Receiver operating characteristic (ROC) curves of DenseNet, ResNet, GoogLeNet, and Majority voting in the test set by Method III.

After training the models, we assessed the system’s performance on the test set of Dataset B. Figure 6 shows that PTC-MAS achieved AUCs ranging from 0.802 to 0.858, with the best performance observed with majority voting. To provide a comprehensive evaluation of PTC-MAS, we calculated additional metrics, as presented in Table 3. The results indicated that ResNet had better $F_{1}$ score, sensitivity, accuracy, PPV, and NPV than the other models.

**Figure 7:**
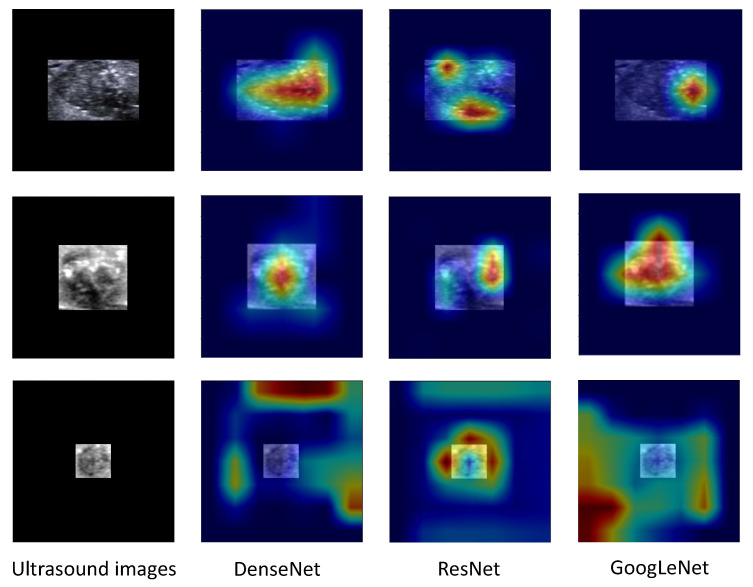
Class Activation Maps of ultrasound images for a nodule with metastasis (**upper rows**, a true positive instance), a nodule without metastasis (**middle rows**, a true negative instance), and false negative instance (**lower rows**).

Moreover, we generated heatmaps using CAM to identify areas of interest for our models. The red and yellow regions in the heatmap correspond to those strongly activated by the neural network, indicating high evaluative significance. Conversely, green and blue regions had weaker evaluative significance. As depicted in Figure 7, we observed that for TP and TN cases the red and yellow regions were primarily located on the nodule itself, rather than the surrounding 0-pixel padding. This finding suggested that our system successfully extracted relevant information from nodules. However, for the FN instance, it seems that the models did not glean valuable information from the ultrasound images.

**Table 3:** Performance of different models on the test set by Method II and Method III.

	DenseNet		ResNet		GoogLeNet		Majority Voting	
	Method II	Method III	Method II	Method III	Method II	Method III	Method II	Method III
AUC (95% CI)	0.736 (0.659–0.803)	0.802 (0.731–0.862)	0.761 (0.685–0.826)	0.837 (0.769–0.892)	0.669 (0.620–0.771)	0.823 (0.753–0.880)	0.759 (0.683–0.824)	0.858 (0.793–0.909)
Sensitivity (95% CI)	0.603 (0.477–0.717)	0.706 (0.581–0.807)	0.676 (0.551–0.782)	0.721 (0.597–0.819)	0.603 (0.477–0.717)	0.721 (0.597–0.819)	0.632 (0.506–0.744)	0.735 (0.612–0.831)
Specificity (95% CI)	0.814 (0.712–0.887)	0.802 (0.670–0.877)	0.756 (0.649–0.839)	0.978 (0.911–0.996)	0.698 (0.588–0.790)	0.837 (0.739–0.905)	0.779 (0.674–0.859)	0.907 (0.820–0.956)
Accuracy	0.721	0.760	0.721	0.864	0.656	0.786	0.714	0.838
$F_{1}$	0.656	0.721	0.681	0.824	0.607	0.748	0.662	0.794
PPV (95% CI)	0.719 (0.583–0.826)	0.738 (0.612–0.836)	0.687 (0.560–0.791)	0.961 (0.854–0.993)	0.612 (0.485–0.726)	0.778 (0.652–0.869)	0.694 (0.561–0.801)	0.862 (0.741–0.934)
NPV (95% CI)	0.722 (0.620–0.806)	0.775 (0.672–0.854)	0.747 (0.641–0.832)	0.816 (0.724–0.883)	0.690 (0.580–0.782)	0.791 (0.691–0.867)	0.728 (0.624–0.813)	0.813 (0.717–0.882)

AUC: the area under the curve, PPV: positive predictive value, NPV: positive predictive value.

### 4.4. Performance of Different Image Extension Methods

To compare the influence of different image cropping methods on PTC-MAS performance, we trained and tested PTC-MAS using various methods on Dataset B with consistent system structures and parameter settings, including learning rate, weight decay, AdamW optimizer, and cross-entropy loss. The only difference was the image cropping method used.

**Figure 8:**
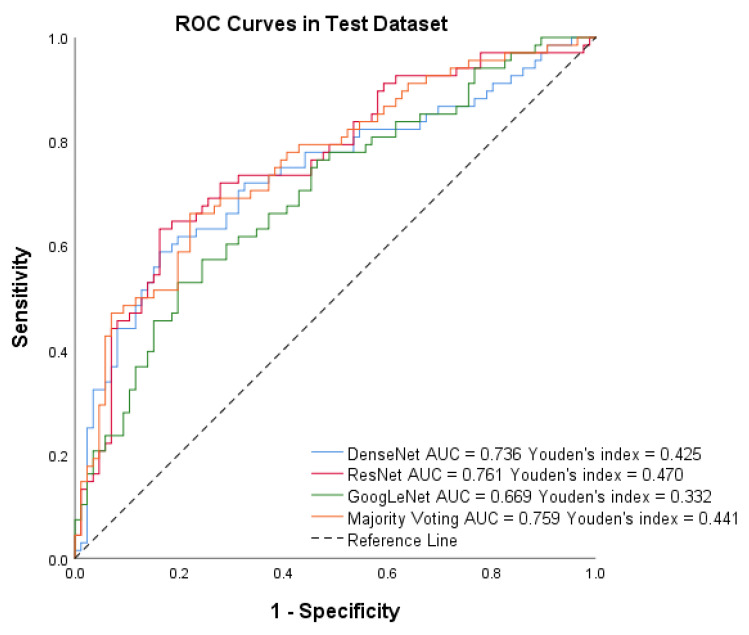
Receiver operating characteristic (ROC) curves of DenseNet, ResNet, GoogLeNet, and Majority voting in the test set using Method II.

For the test dataset, PTC-MAS using Method II achieved AUCs ranging from 0.669 to 0.761, as shown in Figure 8, while those using Method III, achieved AUCs ranging from 0.802 to 0.858, as shown in Figure 6. We also calculated additional metrics such as $F_{1}$ score, sensitivity, accuracy, precision, specificity, PPV, and NPV for the different models using Method II, as presented in Table 3.

**Figure 9:**
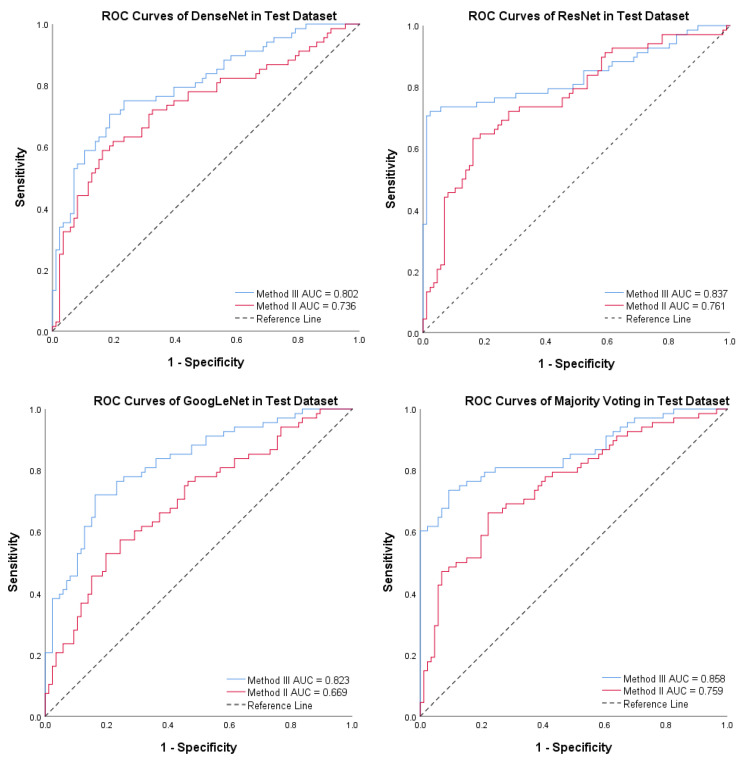
Receiver operating characteristic (ROC) curves for the four models using Method II and Method III. The red line denotes Method II, while the blue line indicates Method III.

To accurately assess the comparative impact of Method II and Method III on PTC-MAS, we employed DeLong’s test to evaluate the differences between the AUCs of the respective ROC curves. Additionally, we generated ROC curves for distinct models utilizing these two methods, as illustrated in Figure 9. As depicted in Table 4, the AUCs of PTC-MAS constructed with Method III were significantly better than those constructed with Method II (*p* < 0.05). Our findings suggest that Method III is more effective in improving the performance of PTC-MAS compared to Method II, as it preserves the relative original size of nodules, which is consistent with recent studies [16,17,18,19].

**Table 4:** The area under the curves (AUCs) of different methods on the test set by Method II and Method III.

	Method III (95% CI)	Method II (95% CI)	*p* Value
DenseNet	0.802 (0.731–0.862)	0.736 (0.659–0.803)	0.0437
ResNet	0.837 (0.769–0.892)	0.761 (0.685–0.826)	0.0253
GoogLeNet	0.823 (0.753–0.880)	0.669 (0.620–0.771)	0.0014
Majority Voting	0.858 (0.793–0.909)	0.759 (0.683–0.824)	0.0001

## 5. Discussion

In this study, we developed an automatic system named PTC-MAS for assessing LNM in primary thyroid cancer using transfer learning to fine-tune DenseNet, ResNet, and GoogLeNet. PTC-MAS is designed to analyze just one ultrasound image of the thyroid cancer, which addresses the challenges associated with ultrasound scanning of cervical lymph nodes and simplifies the input required for the analysis.

PTC-MAS consists of two main parts. In the ROIs acquisition component, our system automatically identifies and crops target nodules, reducing radiologists’ workload, and improving reproducibility by providing a standard ROIs acquisition network. The network achieved high precision and sensitivity, indicating that PTC-MAS accurately extracts ROIs [49,50,51]. In the assessment component, three independent networks achieved excellent classification results. The performance of the network using Method III was superior to that of Method II, suggesting that our networks learn ultrasound image features well. Majority voting improved AUCs compared to individual networks, but ResNet outperformed other individual networks and majority voting, particularly in specificity, accuracy, $F_{1}$ score, PPV, and NPV. This finding may be due to the unbalanced positive and negative samples in our dataset, with the positive to negative sample ratio of 0.79 (441/561). To avoid the impact of data imbalance, AUC was the main index for evaluation. Our system could effectively assess primary thyroid cancer LNM, providing crucial information for subsequent patient treatment and avoiding unnecessary lymph node dissection.

We compared our proposed model with state-of-the-art systems developed by previous researchers. Zhu et al. [33] utilized six different machine learning methods to construct a thyroid cancer LNM assessment model, but their best AUC was only 0.75. Meanwhile, Wang et al. [52] combined clinical factors, B-mode ultrasound, and contrast-enhanced ultrasound features to develop a thyroid cancer LNM assessment model with an AUC of 0.832. In contrast, our proposed system requires only B-mode ultrasound images, simplifying the input and achieving an improved AUC of 0.858. However, it is worth noting that a recent multimodal model [37] combining B-mode and CDFI ultrasound images of cervical lymph nodes with clinical data from patients achieved an impressive AUC of 0.973 for LNM in thyroid cancer on an independent test set. Nonetheless, that study used images of cervical lymph nodes as input, which can be challenging to obtain accurately due to factors such as the expertise of radiologists and interference from gas in the trachea and esophagus. Our proposed system overcomes these limitations and achieves a high AUC, which is a significant advantage of our system.

We also investigated the effect of image cropping on the system, comparing two processing methods. Method III outperformed Method II in both individual networks and majority voting (*p* < 0.05), consistent with prior clinical studies highlighting the importance of the size of thyroid cancer nodules as an independent predictor of LNM [16,17,18,19]. This study has several limitations that should be acknowledged. Firstly, it is a retrospective and single-center study, which may introduce data bias. To enhance the generalizability of our findings, future prospective multicenter studies should be conducted. These studies can involve dynamic imaging and a more extensive range of thyroid cancer types to provide a more comprehensive evaluation of our system’s performance. Secondly, the ultrasound images used in this study were static, and features from multiple cross-sections were not considered. Future studies can incorporate dynamic imaging and analyze features from multiple planes to improve the accuracy of our model. Thirdly, the majority of thyroid cancers included in this study were papillary carcinomas, and there were fewer images of other pathological types of thyroid cancers. A larger sample size with a more diverse range of thyroid cancer types would provide a more comprehensive evaluation of our model’s performance. Fourthly, it is important to note that our system assesses LNM in primary thyroid cancer but does not provide information regarding benign or malignant nodes and cannot localize corresponding lymph nodes. This will be the goal of our follow-up study. Despite these limitations, our proposed system shows promising results and can assist clinicians in patient management and avoid unnecessary neck lymph node dissection.

## 6. Conclusions

We developed PTC-MAS, an automated system that accurately assesses primary thyroid cancer LNM using a single ultrasound image of a thyroid nodule. Our approach incorporates a novel image cropping method that aligns with previous clinical studies and preserves important nodule size features. Notably, our approach retains nodule size features, a novel contribution in this field. Compared to existing methods, PTC-MAS offers several advantages, such as automatic and precise ROIs extraction, reducing radiologists’ workload. By combining individual networks, our system achieves excellent performance, outperforming previous studies. We believe that PTC-MAS has the potential to improve preoperative assessment of primary thyroid cancer LNM and ultimately contribute to better patient outcomes.

## Data Availability

Data are unavailable due to privacy or ethical restrictions.

## Sample Availability

The relevant code is available from the authors.

## Abbreviations

The following abbreviations are used in this manuscript:

LNM	Lymph Node Metastasis
PTC-MAS	Primary Thyroid Cancer Lymph Node Metastasis Assessment System
ROI	Region of Interest
YOLOS	YOLO Thyroid Nodule Recognition System
AUC	Receiver Operating Characteristic Curve
AJCC	The American Joint Committee on Cancer
SAHCMU	The Second Affiliated Hospital of Chongqing Medical University
FNA	Fine-Needle Aspiration
ACR	American College of Radiology
TI-RADS	Thyroid Imaging Reporting and Data System
PACS	The Picture Archiving and Communication System
CAM	Class Activation Maps
TP	True Positives
FP	False Positives
TN	True Negatives
FN	False Negatives
PPV	Positive Predictive Value
NPV	Negative Predictive Value
CI	Confidence Interval
